# Influence of breed on selected quality parameters of fresh goat meat

**DOI:** 10.5194/aab-63-219-2020

**Published:** 2020-07-14

**Authors:** Snežana Ivanović, Marija Pavlović, Ivan Pavlović, Aleksandra Tasić, Jelena Janjić, Milan Ž. Baltić

**Affiliations:** 1 Scientific Veterinary Institute of Serbia, Belgrade, 11000, Serbia; 2 Faculty of Veterinary Medicine, University of Belgrade, Belgrade, 11000, Serbia

## Abstract

The potential of goats to produce a high-quality meat is mainly reflected in their healthy fats, low calorie intramuscular fats, saturated fats, and, especially, their high ratios of unsaturated (UFA) and saturated (SFA) fatty acids, as well as hypocholesterolemic and hypercholesterolemic fatty acids. The aim of this study was to collect and compare meat quality parameters for domestic Balkan, Alpine and Saanen goats of the same age. Samples for all tests were taken from *musculus gluteus superficialis*. Chemical composition, pH value, fatty acid composition, content of volatile compounds, color and overall sensory quality (appearance, texture and smell) were determined. In chemical composition, moisture, fat, protein and ash varied significantly between each of the examined groups as opposed to pH values. Furthermore, among all the examined groups a significant difference was found for fatty acids and volatile compounds. Determined ratio of polyunsaturated fatty acids (PUFAs) to SFAs was 0.089, 0.085 and 0.071 for Balkan, Alpine and Saanen goat meats, respectively. Regarding that ratio, Saanen goat meat had the most favorable characteristics. Saanen goat meat showed the highest nutritional value. On the other hand, Balkan goat meat had the lowest intramuscular fat content. Measurements of the meat color from all three groups, as well as overall acceptability, showed significant differences between breeds. Obtained results point to the impact of breed on chemical composition and fatty acid profile of goat meat.

## 1 Introduction

In most countries, goats are the most spread ruminants of all domesticated ruminants due to their ability to adapt themselves which is especially expressed in difficult climatic conditions (Tefiel et al., 2018). The number of goats in the world was, in 2018, over a billion – 1 046 146 964 (FAO, 2020) – and in Europe there were 10 433 360 goats in 2019 (Eurostat, 2020). According to the Statistical Office of the Republic of Serbia (2019) in 2018, 195 934 goats were reared in Serbia. The local goat population is mainly located in small rural areas, and they are generally reared in very small herds on single-family farms.

Although the number of goats in Serbia is constantly increasing (Žujović et al., 2009), goat meat is not traditionally consumed due to consumers' habit, and the meat cannot easily be found in retail stores.

Of the total number of goats registered in Serbia, the Alpine goat is the most dominant accounting for 87 % of animals, followed by two local breeds: Balkan goats and Serbian White goats. Alpine goats are widespread throughout different regions of Serbia, as well as globally. The domestic Balkan goat is a primitive goat breed in Serbia. This is the indigenous goat breed that is listed in the national program for the protection and conservation of animal genetic resources. The pure bred Saanen goat, an ideal white dairy goat breed with high productivity, is mainly raised for milk production (Žujović et al., 2009, 2011).

Producers and consumers are showing increasing interest in goat meat since the animals have very small amounts of subcutaneous and intramuscular fat. Compared to sheep of a similar age, their carcasses are very lean (Stanišić et al., 2012). Nutritionally, goat meat is an important source of high-quality protein, healthy fats, low calorie intramuscular fats, saturated fats and sodium. In addition, goat meat has high levels of iron, potassium and essential amino acids (McMillin and Brock, 2005; Givens et al., 2006; Lee et al., 2008; Horcada et al. 2012).

Fatty acids play an important role in human nutrition. All unsaturated fatty acids (UFAs) plus stearic fatty acids (UFAs + C18:0) are categorized as desirable fatty acids (DFAs), while all saturated fatty acids (SFAs) minus stearic fatty acids (SFAs - C18:0) are considered hypercholesterolemic or undesirable fatty acids (OFAs) (Solaiman et al., 2011). Unsaturated fatty acids (UFAs) have a hypocholesterolemic effect. However, not all SFAs have the same effects. Lauric (C12:0), myristic (C14:0) and palmitic (C16:0) acids are hypercholesterolemic, while saturated stearic (C18:0) acid does not increase blood cholesterol levels and is considered neutral (Banskalieva et al., 2000). In general, the high ratio of UFA / SFA and DFA / OFA in goat meat demonstrates the potential of goats to produce meat with high nutritional value (Brzostovski et al., 2008; Pena et al., 2009). Meat quality can be influenced by several factors such as slaughter age, breed, genotype, gender, diet and treatment during slaughter (Costa et al., 2008; Toplu et al., 2013; Čobanović et al., 2019). On the other hand, appearance, tenderness, taste and juiciness are important categories that affect the consumers' acceptability of goat meat (Madruga et al., 2008; Silva et al., 2011).

The aim of this study was to examine some physical, chemical and sensory characteristics in goat meat of different breeds.

## 2 Materials and methods

### 2.1 Ethics statement

All the experimental procedures and animal handling used in this study were in accordance with guidelines of the European Community (Directive86/609/EEC) and guidelines of the Ethics Committee of the Faculty of Veterinary Medicine, University of Belgrade, Serbia.

### 2.2 Animals and sampling

Balkan goats, Alpine goats and Saanen goats were studied: 120 individuals in total, 40 of each breed. All goats were raised during the same period and were about 4 years old at slaughter, which occurred during August–September. All goats were selected from private farms in the mountain region Stara Planina (height 1000–1500 ma.s.l.), Serbia. Animals had ad libitum access to water. The goats' winter diets consisted of hay collected from natural pastures (3.5 $\mathrm{kg}\;d^{-1}$ per animal) and concentrate (0.25 $\mathrm{kg}\;d^{-1}$ per animal). In summer, the goats were pastured and fed with concentrate (0.25 $\mathrm{kg}\;d^{-1}$). The winter hay and summer pasture originated from natural meadows in the region. Plants were determined and their names harmonized with the nomenclature according to the published literature (Lakusić and Cetković, 2007; Stevanović, 2012). Botanical composition of hay and pasture was as follows: *Arrhenatheretum elatioris*, *Festuco–Chrysopogonetum*, *Danthonietum calycinae*, *Medicago falcate–Festucetum rubrae*, *Trifolio campestre–Agrostietum vulgaris*, *Festuco vallesiacae* and *Agrostieutum vulgaris* with the predomination of family *Poaceae* (*Arrhenatherum elatius*, *Dactylis glomerata*, *Festuca pratensis Huds. i Lolium perenne* and family *Fabaceae* (*Lotus corniculatus*, *Trifolium repens*, *Lathyrus pratensis*, *Trifolium montanum*, *Trifolium campestre*). The concentrate feedstuff was composed of maize meal, wheat bran, sodium chloride and premix. This latter consisted of vitamins – A, D3, E, K3, C, B1, B2, B12, Niacin and Ca-pantothenate – and minerals – Fe, Cu, Mn, Zn, J, Se, Co and Mg. Complete feed mixture was formulated to meet or exceed the requirements set by the National Research Council (2007).

Preslaughter live weights of goats were recorded, and the animals were slaughtered in 3 consecutive days in the slaughterhouse at the Institute for Animal Husbandry, Belgrade, using captive-bolt stunning followed by the cutting of the carotid arteries and jugular veins. Carcasses were processed using standard industrial procedures and cooled at 4 ${}^{\circ }$C for 48 h. The 120 samples of goat meat – *musculus gluteus superficialis* – were collected during the trial. Individual muscle samples served as experimental units for analyses; hence, 40 samples for each examined breed were included in the study.

### 2.3 Chemical composition of meat

Within 48 h of slaughter, muscles were excised from the carcasses and packed into the polyethylene bags to protect the meat against drying. After packing and before analysis, meat samples were stored at -20 ${}^{\circ }$C. All analyses were conducted within 1 month of packing. Meat samples were thawed before examination at refrigerator temperature (+2 ± 2 ${}^{\circ }$C). After defrosting and before analysis, samples were weighed to around 110 g, and the thickness of the samples was 15 mm.

The proximal composition, pH, fatty acid content, volatile compound content, color and sensory acceptability were determined in the *m. gluteus superficialis*. Moisture content was determined by ISO 1442 (1998), fat content by ISO 1433 (1992) and ash content by ISO 936 (1999). The protein content was calculated from the nitrogen content multiplied by 6.25 using ISO 937 (1992), and pH was determined by ISO 2917 (2004).

### 2.4 Fatty acids

The AOAC 996.06 (2001) method was applied to the lipid extraction from the tissue. After the lipid hydrolysis, the fatty acids were esterified to methyl esters, evaporated to dryness in a stream of nitrogen and stored at -18 ${}^{\circ }$C. An analysis of fatty acid methyl esters (FAMEs) was performed by an internal standard method using a gas chromatograph (GC6890N, Agilent Tech., USA) with column DB-23 (60 m × 0.25 mm ID, 0.15 µm) and peak areas and retention times were compared with a standard mix of FAME 37 (Supelco, USA). The conditions of the analyses are as follows: detector temperature – 250 ${}^{\circ }$C; injector temperature – 225 ${}^{\circ }$C; column temperature – 200 ${}^{\circ }$C; carrier gas – helium; and carrier gas flow rate – 50 $\mathrm{mL}\;\min^{-1}$. Obtained data for the composition of fatty acids were expressed in percentages by weight of the identified total fatty acids.

### 2.5 Volatile compounds

Volatile compound analyses were conducted using the extraction procedure from Likens-Nickerson (1964) followed by gas chromatography–mass spectrometry (GCMS) according to ISO 15303 (2001) using an GCMS-QP2010 Ultra (EIMS, electron ionization mass spectrometry; electron energy – 70 eV, scan range – 30–350 amu and scan rate – 3.99 $\mathrm{scans}\;s^{-1}$) with a SUPELCOWAX${}^{\text{®}}$  10 Capillary GC Column (30 m × 0.25 mm ID, particle size 0.25 µm). The carrier gas was helium with a flow rate of 1 $\mathrm{mL}\;\min^{-1}$, and the injection temperature was 200 ${}^{\circ }$C. The oven temperature was programmed to initially hold for 10 min at 40 ${}^{\circ }$C and then to heat from 40 to 120 ${}^{\circ }$C at a rate of 3 ${}^{\circ }C\;\min^{-1}$ and subsequently at a rate of 10 ${}^{\circ }C\;\min^{-1}$ from 120 to 250 ${}^{\circ }$C, at which point it was held for another 5 min. Identification of the peaks was based on a comparison of their mass spectra with the spectra of the WILEY library and, in some additional cases, on a comparison of their retention times with those of standard compounds.

### 2.6 Color

Meat samples were bloomed for 30 min before the color measurement. CIE L*a*b* (CIE, 1986) color coordinates of meat were determined using a Minolta CR 400 Chroma Meter (Minolta Co. Ltd., Osaka, Japan) in D-65 lighting with a standard angle of 2${}^{\circ }$ of shelter and an 8 mm aperture measuring head. CIE L*a*b* measurements are reported as mean values: L* (lightness), a* (redness) and b* (yellowness).

### 2.7 Sensory analysis

Sensory analyses were carried out in the sensory laboratory equipped with individual booths. Each booth is made up of three walls on each side in order to prevent panelists influencing each other. All booths were provided with florescent lights to mask color differences between the samples. Sensory tests were performed at room temperature (22–24 ${}^{\circ }$C). After a cooling period (at 4 ${}^{\circ }$C) of 24 h, all tested samples were marked by random three-digit numbers. All meat samples were subjected to equal heat treatment in an electric oven at 170 ${}^{\circ }$C until a constant internal temperature of 80 ${}^{\circ }$C was reached. The meat samples were cut to an approximate size of 2.5 cm × 2.5 cm × 2.5 cm. The samples were served individually to each panelist, in plastic bowls, at an internal meat temperature of 60 ± 5 ${}^{\circ }$C immediately after heat treatment. Tap water was provided to rinse the palate between samples.

The order of presentation of the samples was randomized for 20 trained panelists. The selection of panelists was conducted according to ISO standards (ISO 8586, 2012, 2015).

Overall acceptability was evaluated based on appearance, texture and aroma. For the evaluation, the scoring range from one to five was used, with the possibility of assigning half and quarter points. For each selected quality property, the coefficient of importance is determined in order to correct the given estimate by the multiplication of means. The coefficients of importance were chosen according to the influence of certain properties on the overall quality (4 for surface color, 3 for visual evaluated structure, 3 for palpatory evaluated firmness and 10 for olfactory evaluated odor), and their sum is 20. By combining individual scores, a complex indicator is obtained which represents the overall sensory quality and is expressed as a percent of the maximum possible quality (maximum possible quality is 100 %). By dividing this value with a set of coefficients of importance, a weighted average score is obtained, which also represents the total sensory quality of the tested samples of goat meat.

The scores are as follows:

- 1.00: very pronounced errors;
- 2.00: clearly expressed mistakes;
- 3.00: noticeable deviations;
- 4.00: minor deviations;
- 5.00: fully meets the quality requirements.

### 2.8 Statistical analysis

Data obtained in this study were analyzed using GraphPad Prism 6.0 software (GraphPad Software Inc., San Diego, CA, USA). All values are expressed as means and standard deviation. The differences between means were compared by one-way analysis of variance (one-way ANOVA) at the level of significance of 95 %. The significance of differences between mean values in groups was compared with Tukey's test at the level of significance of 95 %.

## 3 Results

### 3.1 Live weight, chemical composition and pH value of meat

Table 1 shows the live weights and the chemical composition and pH of the goat meat according to goat breed.

**Table 1:** Goat live weight and *m. gluteus superficialis* proximal composition and pH (n=40 per breed).

Parameter	Balkan goat	Alpine goat	Saanen goat	P value (ANOVA)
Live weight (kg)	42.45 ± 2.10${}^{a}$	44.30 ± 2.40${}^{b}$	47.23 ± 2.80${}^{c}$	0.013
Carcass weight (kg)	19.08 ± 0.11${}^{a}$	19.99 ± 0.08${}^{b}$	21.31 ± 0.19${}^{c}$	0.018
Moisture (%)	74.32 ± 0.14${}^{a}$	74.55 ± 0.18${}^{b}$	74.77 ± 0.17${}^{c}$	0.028
Fat (%)	3.82 ± 0.16${}^{a}$	3.90 ± 0.15${}^{b}$	4.05 ± 0.20${}^{c}$	0.012
Protein (%)	19.45 ± 0.04${}^{a}$	19.52 ± 0.05${}^{b}$	19.82 ± 0.07${}^{c}$	0.037
Ash (%)	0.96 ± 0.02${}^{a}$	0.98 ± 0.03${}^{b}$	1.01 ± 0.03${}^{c}$	0.027
pH after 24 h	5.68 ± 0.04${}^{\text{ns}}$	5.66 ± 0.05${}^{\text{ns}}$	5.65 ± 0.03${}^{\text{ns}}$	0.18

${}^{\text{ns}}$ No significant difference.${}^{\text{a, b, c}}$ Means within the same row with different superscripts differ significantly (P<0.05).

**Table 2:** Fatty acid composition of fresh goat meat (% of total fatty acids) (*m. gluteus superficialis*) (n=40 per breed).

Parameter	Balkan goat	Alpine goat	Saanen goat	P value (ANOVA)
Capric acid (C10:0)	0.51 ± 0.04${}^{a}$	0.60 ± 0.05${}^{b}$	0.64 ± 0.04${}^{c}$	0.011
Lauric acid (C12:0)	0.91 ± 0.03${}^{a}$	1.30 ± 0.04${}^{b}$	1.23 ± 0.03${}^{c}$	0.013
Myristic acid (C14:0)	10.02 ± 0.05${}^{a}$	10.31 ± 0.05${}^{b}$	10.52 ± 0.06${}^{c}$	0.02
Pentadecanoic acid (C15:0)	1.34 ± 0.02${}^{a}$	1.52 ± 0.03${}^{b}$	1.52 ± 0.02${}^{c}$	0.017
Palmitic acid (C16:0)	25.01 ± 0.10${}^{a}$	25.97 ± 0.13${}^{b}$	26.78 ± 0.14${}^{c}$	0.013
Margaric acid (C17:0)	0.19 ± 0.01${}^{a}$	0.23 ± 0.02${}^{b}$	0.29 ± 0.01${}^{c}$	0.014
Stearic acid (C18:0)	14.70 ± 0.09${}^{a}$	14.11 ± 0.10${}^{b}$	15.09 ± 0.10${}^{a}$	0.023
Arachidic acid (C20:0)	0.52 ± 0.04${}^{a}$	0.63 ± 0.05${}^{b}$	0.75 ± 0.05${}^{c}$	0.017
Pentadecenoic acid (C15:1)	0.18 ± 0.02${}^{a}$	0.14 ± 0.01${}^{b}$	0.10 ± 0.01${}^{c}$	0.015
Palmitoleic acid (C16:1)	2.30 ± 0.03${}^{a}$	2.42 ± 0.02${}^{b}$	2.13 ± 0.02${}^{c}$	0.011
Eicosenoic acid (C20:1)	0.36 ± 0.02${}^{a}$	0.23 ± 0.02${}^{b}$	0.12 ± 0.01${}^{c}$	0.018
Heptadecenoic acid (C17:1nXc)	0.24 ± 0.02${}^{a}$	0.16 ± 0.01${}^{b}$	0.10 ± 0.01${}^{c}$	0.012
Elaidic acid (C18:1n9t)	0.35 ± 0.03${}^{a}$	0.28 ± 0.02${}^{b}$	0.18 ± 0.03${}^{c}$	0.019
Oleic acid (C18:1n9c)	36.92 ± 0.14${}^{a}$	36.55 ± 0.13${}^{a}$	36.01 ± 0.11${}^{b}$	0.027
Linolelaidic acid (C18:2n6t)	0.47 ± 0.03${}^{a}$	0.40 ± 0.04${}^{b}$	0.30 ± 0.05${}^{c}$	0.016
Linoleic acid (C18:2n6c)	2.98 ± 0.04${}^{a}$	3.01 ± 0.04${}^{b}$	2.72 ± 0.05${}^{c}$	0.021
Alpha-linolenic acid (C18:3n6)	1.30 ± 0.02${}^{a}$	1.22 ± 0.01${}^{a}$	1.01 ± 0.01${}^{b}$	0.028

${}^{\text{a, b, c}}$ Means within the same row with different superscripts differ significantly (P<0.05).

The Saanen goat was heavier, on average, than the Balkan and Alpine goat (Table 1). The mean contents of moisture, fat, protein and ash all differed between the goat breeds (P<0.05). However, the mean meat pH post rigor did not differ between the three goat breeds (P>0.05).

### 3.2 Fatty acid composition of goat meat

The fatty acid composition of *m. gluteus superficialis* from Balkan goat, Alpine goat and Saanen goat is presented in Table 2. All determined fatty acids differed significantly between three examined breeds (P<0.05) except oleic and alpha-linolenic acid which did not differ between Balkan and Alpine goat meats (P>0.05) and stearic acid which did not differ between Balkan and Saanen goats (P>0.05).

The sums and ratios of fatty acids in the goat meat is presented in Table 3. The ratio of unsaturated to saturated fatty acids in Balkan goats was 0.85, in Alpine goats it was 0.81, and in Saanen goats it was 0.82. Calculated ratios, as well as nutritional value, were significantly different between examined breeds (P<0.05).

**Table 3:** Indicators of the nutritional value and health benefits of fat determined based on an analysis of the fatty acid profile of goat meat (n=40 per breed).

Indicator${}^{1}$	Balkan goat	Alpine goat	Saanen goat	P value (ANOVA)
ΣSFA	53.20 ± 1.39	54.67 ± 0.79	56.83 ± 2.02	0.061
ΣUFA	45.10 ± 1.75	44.38 ± 1.54	46.67 ± 1.44	0.07
ΣMUFA	40.35 ± 1.94	39.75 ± 2.01	38.64 ± 1.43	0.064
ΣPUFA	4.75 ± 1.04	4.63 ± 1.51	4.03 ± 1.39	0.08
UFA/SFA	0.85 ± 0.01${}^{a}$	0.81 ± 0.008${}^{b}$	0.82 ± 0.007${}^{c}$	0.021
MUFA/SFA	0.76 ± 0.02${}^{a}$	0.73 ± 0.03${}^{a}$	0.68 ± 0.01${}^{b}$	0.023
PUFA/SFA	0.089 ± 0.02${}^{a}$	0.085 ± 0.02${}^{b}$	0.071 ± 0.01${}^{c}$	0.017
DFA	59.80 ± 1.28${}^{a}$	58.49 ± 1.24${}^{a}$	61.76 ± 1.58${}^{b}$	0.022
OFA	38.50 ± 1.02${}^{a}$	40.56 ± 1.15${}^{b}$	41.74 ± 0.63${}^{b}$	0.034
EFA	4.75 ± 0.52${}^{a}$	4.63 ± 0.79${}^{b}$	4.03 ± 0.16${}^{c}$	0.024
Nutritional value${}^{*}$	16.19 ± 0.29${}^{a}$	15.53 ± 0.35${}^{b}$	16.44 ± 0.11${}^{c}$	0.019

SFA – Saturated fatty acid; UFA – Unsaturated fatty acid; MUFA – monounsaturated fatty acid; PUFA – polyunsaturated fatty acid; DFA – hypocholesterolemic fatty acid (UFA + C18:0); OFA – hypercholesterolemic fatty acid (SFA – C18:0); EFA – essential fatty acid (C18:2 + C18:3).${}^{1}$ Calculations of all indicators in Table 6 were made according to Daszkiewicz and Mesinger (2018).${}^{*}$ Nutritional value was calculated according to the equation C18:0 + C18:1 / C16:0.${}^{\text{a, b, c}}$ Means within the same row with different superscripts differ significantly (P<0.05).

### 3.3 Volatile substances in goat meat

Table 4 shows the presence of specific volatile substances in *m. gluteus superficialis* in the three goat breeds, Balkan goat, Alpine goat and Saanen goat. The group of aldehydes was the most common groups of compounds identified in the analyzed goat meats. Among the aldehydes, 15.77 ± 0.10 $µg\;{\mathrm{kg}}^{-1}$ of hexanal was detected in Balkan goat meat, 15.89 ± 0.12 $µg\;{\mathrm{kg}}^{-1}$ in Alpine goat meat and 16.12 ± 0.14 $µg\;{\mathrm{kg}}^{-1}$ in Saanen goat meat. The second most common group of compounds was the ketones. The predominant ketones in our fresh goat meat were 2-butanone and 2,3-butanedione. Among the aromatic hydrocarbons we tested for, only methylbenzene was detected (Table 4). No phenols were detected in the goat meat examined. Also, only small quantities of organic acids were present in the meat. Overall, Balkan goat meat contained fewer volatile substances than meat from Alpine and Saanen goats.

**Table 4:** Volatile substances in fresh *m. gluteus superficialis* meat ($µg\;{\mathrm{kg}}^{-1}$) according to goat breed (n=40 per breed).

Volatile substance ($µg\;{\mathrm{kg}}^{-1}$)	Balkan goat	Alpine goat	Saanen goat	P value (ANOVA)
Aldehydes				
3-Methylbutanal	1.12 ± 0.03${}^{a}$	1.18 ± 0.03${}^{b}$	1.26 ± 0.04${}^{c}$	0.017
Pentanal	2.89 ± 0.05${}^{a}$	3.10 ± 0.05${}^{b}$	3.29 ± 0.06${}^{c}$	0.013
Hexanal	15.77 ± 0.10${}^{a}$	15.89 ± 0.12${}^{b}$	16.12 ± 0.14${}^{c}$	0.016
Heptanal	2.16 ± 0.08${}^{a}$	2.27 ± 0.09${}^{b}$	2.43 ± 0.10${}^{c}$	0.019
Benzaldehyde	0.17 ± 0.02${}^{a}$	0.23 ± 0.02${}^{b}$	0.31 ± 0.03${}^{c}$	0.018
Octanal	1.55 ± 0.04${}^{a}$	1.69 ± 0.04${}^{b}$	1.82 ± 0.05${}^{c}$	0.013
Nonanal	2.85 ± 0.07${}^{a}$	2.99 ± 0.07${}^{b}$	3.16 ± 0.08${}^{c}$	0.019
Ketones				
2,3-Butanedione	0.22 ± 0.03${}^{a}$	0.27 ± 0.03${}^{b}$	0.37 ± 0.04${}^{c}$	0.015
2-Butanone	3.40 ± 0.08${}^{a}$	3.52 ± 0.09${}^{b}$	3.59 ± 0.10${}^{c}$	0.02
2-Pentanone	0.08 ± 0.01${}^{a}$	0.13 ± 0.01${}^{b}$	0.19 ± 0.02${}^{c}$	0.019
3-Hydroxy-2-butanone	0.12 ± 0.02${}^{a}$	0.18 ± 0.03${}^{b}$	0.26 ± 0.03${}^{c}$	0.023
2-Heptanone	0.22 ± 0.03${}^{a}$	0.29 ± 0.03${}^{b}$	0.35 ± 0.04${}^{c}$	0.021
2,3-Octanedione	0.19 ± 0.02${}^{a}$	0.24 ± 0.02${}^{b}$	0.32 ± 0.03${}^{c}$	0.017
Heterocyclic compounds				
2,6-Dimethylpyrazine	0.07 ± 0.02${}^{a}$	0.11 ± 0.03${}^{b}$	0.18 ± 0.03${}^{c}$	0.016
Aromatic hydrocarbons				
Methylbenzene	0.09 ± 0.03${}^{a}$	0.14 ± 0.03${}^{b}$	0.20 ± 0.03${}^{c}$	0.019
Phenols${}^{\text{nd}}$				
Alcohols				
1-Penten-3-ol	0.15 ± 0.03${}^{a}$	0.25 ± 0.03${}^{b}$	0.36 ± 0.04${}^{c}$	0.021
2-Pentanol	0.12 ± 0.02${}^{a}$	0.19 ± 0.03${}^{b}$	0.27 ± 0.03${}^{c}$	0.019
3-Methyl-1-butanol	0.10 ± 0.02${}^{a}$	0.16 ± 0.03${}^{b}$	0.22 ± 0.03${}^{c}$	0.021
1-Pentanol	1.04 ± 0.07${}^{a}$	1.17 ± 0.07${}^{b}$	1.25 ± 0.08${}^{c}$	0.014
Furfurol	0.10 ± 0.03${}^{a}$	0.18 ± 0.03${}^{b}$	0.29 ± 0.04${}^{c}$	0.013
1-Octen-3-ol	0.99 ± 0.03${}^{a}$	1.11 ± 0.04${}^{b}$	1.25 ± 0.04${}^{c}$	0.016
Organic acids				
Acetic acid	0.18 ± 0.04${}^{a}$	0.28 ± 0.04${}^{b}$	0.37 ± 0.05${}^{c}$	0.017
Butanoic acid	0.55 ± 0.06${}^{a}$	0.63 ± 0.08${}^{b}$	0.74 ± 0.10${}^{c}$	0.021
3-Methyl-butanoic acid	0.06 ± 0.02${}^{a}$	0.14 ± 0.03${}^{b}$	0.19 ± 0.03${}^{c}$	0.022
Alkanes				
Hexane	0.21 ± 0.03${}^{a}$	0.24 ± 0.03${}^{b}$	0.31 ± 0.05${}^{c}$	0,023
Heptane	0.12 ± 0.03${}^{a}$	0.17 ± 0.03${}^{b}$	0.23 ± 0.03${}^{c}$	0,027
Octane	0.73 ± 0.05${}^{a}$	0.78 ± 0.05${}^{b}$	0.89 ± 0.06${}^{c}$	0.019
Nonane	0.10 ± 0.03${}^{a}$	0.15 ± 0.03${}^{b}$	0.20 ± 0.04${}^{c}$	0.021
Alkenes				
1-Octene	0.12 ± 0.03${}^{a}$	0.19 ± 0.04${}^{b}$	0.28 ± 0.04${}^{c}$	0.017

${}^{\text{nd}}$ Not detected.${}^{\text{a,b,c}}$ Means within the same row with different superscripts differ significantly (P<0.05).

### 3.4 Color of goat meat expressed in CIE L*a*b* system

Color parameters (L*, a*, b*) of goat leg meat derived from *m. gluteus superficialis* are presented in Table 5. A statistically significant difference (P<0.05) occurred between the color of meat originating from Balkan and Saanen goats but not between Balkan and Alpine goats (P>0.05) for all color parameters.

**Table 5:** Color of fresh goat meat following the CIE L*a*b* system (n=40 per breed).

Parameter	L*	a*	b*
Balkan goat	31.66 ± 1.11${}^{a}$	18.43 ± 0.65${}^{a}$	3.88 ± 0.30${}^{a}$
Alpine goat	31.99 ± 1.32${}^{\text{a,b}}$	18.63 ± 0.72${}^{\text{a,b}}$	3.98 ± 0.40${}^{\text{a,b}}$
Saanen goat	32.41 ± 1.47${}^{b}$	18.87 ± 0.83${}^{b}$	4.13 ± 0.52${}^{b}$
P value (ANOVA)	0.031	0.029	≤0.033

L* – lightness; a* – redness; b* – yellowness.${}^{\text{a, b}}$ Means within the same column with different superscripts differ significantly (P<0.05).

### 3.5 Sensory evaluation of goat meat

Comparisons of the overall sensory evaluation of meat from different goat breeds are shown in Table 6. No statistically significant difference was detected in the overall acceptability of Balkan and Alpine goat meats or of Alpine and Saanen goat meats (P>0.05). There was a statistically significant difference (P<0.05) in the acceptability of fresh Balkan and Saanen goat meats for the parameters of surface color and olfactory evaluated odor, as well as for average score. Balkan goat meat was more acceptable compared to the other two examined breeds.

**Table 6:** Sensory evaluation of goat meat.

	Attributes				Percentage of maximal possible quality	Weighted average
	Appearance	Texture		Flavor		
	Surfacecolor	Visuallyevaluatedstructure	Palpatory evaluated firmness	Olfactoryevaluatedodor		
	Coefficient of importance					
	4	3	3	10	100	100/20
Balkan goat	18.60 ± 0.28${}^{a}$	14.70 ± 0.25	13.80 ± 0.23	47.50 ± 0.32${}^{a}$	94.60	4.73${}^{a}$
Alpine goat	19.00 ± 0.25${}^{\text{a,b}}$	13.80 ± 0.18	13.95 ± 0.18	46.00 ± 0.13${}^{\text{a,b}}$	92.75	4.64${}^{\text{a,b}}$
Saanen goat	18.40 ± 0.28${}^{b}$	13.80 ± 0.16	13.20 ± 0.39	44.60 ± 0.20${}^{b}$	90.00	4.50${}^{b}$
P value (ANOVA)	0.019	0.056	0.067	0.024	0.07	*0.031*

${}^{\text{a, b}}$ Means within the same column with different superscripts differ significantly (P<0.05).

## 4 Discussion

Meat quality can be affected by numerous factors: breed, gender, productivity, adaptation to stress, environment, management, nutrition, live weight and carcass weight at slaughter (McMillin, and Brock, 2005; Assan, 2015; Wattanachant, 2018). In the current study, the live weights of the three goat breeds, with Saanen goat being the heaviest of the three breeds, agree with the characteristics of the individual breeds given in previous studies (Dhanda et al., 2003; Memiši and Stanišić, 2014; Ivanović and Pavlović, 2015; Rojo-Rubio et al., 2016).

The percentages of moisture (74.32 %) and fat (3.82 %) in the Balkan goat meat in the current study were not in accordance with earlier studies on Bunte Deutsche Edelziege and Balkan goat meats (Ivanović et al., 2011), Serbian White goat and Balkan goat (Ivanović et al., 2014), or Balkan goats reared in a mountainous area (Ivanović et al., 2016). The protein and ash contents and pH of Balkan goat meat (19.45 %, 0.96 % and 5.68, respectively) were consistent with those in previous studies (Ivanovic et al., 2011, 2014, 2016).

Wattanachant et al. (2008) examined the quality characteristics of raw goat meat obtained from different ages and breeds of goats and proved significant differences between experimental groups. Numerous studies proved an influence of goat breed on meat quality, as well as different genotypes of the same breed (Dhanda et al., 1999; Peña et al., 2009; Brzostowski et al., 2008; Horcada et al., 2012; Özcan et al., 2015). Similar conclusions have been obtained in the current study, in which differences in meat quality between indigenous breeds and breeds improved by selection were examined.

In the current study, the most prevalent fatty acid in all examined breeds was oleic followed by palmitic, stearic and myristic acids. The highest content of oleic acid was determined in meat obtained from Balkan goat (36.92 %) followed by Alpine (36.55 %) and Saanen (36.01 %) goats. Regarding palmitic acid, the most prevalent content was in meat from Saanen goat (26.78 %) followed by Alpine (25.79 %) and Balkan (25.01 %) goats. The determined amounts of stearic acid were 15.09 %, 14.70 % and 14.11 % for Saanen, Balkan and Alpine goats, respectively. The concentration of linoleic acid, an essential fatty acid, was the highest in the gluteus muscles of Alpine goat and the lowest in the Saanen goat.

These results were not the same as in earlier research (Ivanović et al., 2012, 2014) as the breed and method of raising goats were different. During 2012, the fatty acid composition of Bunte Deutsche Edelziege goats which were 3 and 6 years old was investigated. In 2014, the difference in meat quality of Serbian White and Balkan goats, both grown in mountainous areas, was examined (Ivanović et al., 2014). A statistically significant difference in fatty acids was found between these two breeds. In the current study, Balkan goat meat quality parameters were in agreement with the results obtained in previous studies of Balkan goats reared in a mountainous region (Ivanović et al., 2016).

The breed is a source of variation for fat deposition, total intramuscular fat content and fatty acid profile of goat meat (Ding et al., 2010; Horcada et al., 2012; Assan, 2015; Wattanachant, 2018). Dhanda et al. (1999) have shown a 10 to 13 % difference in total fat content in different goat breeds with no significant difference in carcass weight. The influence of breed on the fatty acid profile of adipose tissue has been reported (Dhanda et al., 2003). Özcan et al. (2015) have shown the differences in levels of some fatty acids but not in the ratios of polyunsaturated fatty acids (PUFAs) to saturated fatty acids (PUFAs / SFAs) or n-6 / n-3. In the current study, all determined fatty acids (P<0.05) varied significantly, which points to the impact of breed on the fatty acid profile of meat due to large differences between native breeds and breeds improved by selection. It is important to point out that breed and genotype can not be observed as a single deciding factor for the fatty acid profile of meat, but they are associated with other factors like nutrition, management, age, gender etc. (Wattanachant, 2018).

Among the fatty acids, not all have the same effect on human health. Lauric (C12:0), myristic (C14:0) and palmitic (C16:0) acids are thought to exert atherogenic effects which can thus increase the concentration of low-density lipoprotein (LDL) cholesterol in blood (Howell et al, 1997). Myristic acid has the most adverse effect on the cardiovascular system, which is 4 times more pronounced compared to lauric and palmitic acids (Hegsted et al., 1959). On the other hand, stearic acid is considered to be neutral.

In the current study, the content of lauric, myristic and palmitic acids was the lowest in Balkan goat meat. The highest content of stearic acid was measured in Saanen goat meat, but it was not significantly higher compared to Balkan goat. Thus, the significantly lower content of myristic, palmitic and lauric acids along with determined levels of stearic acid observed in this study point to the greater health benefits of Balkan goat meat over meat obtained from the other two examined breeds.

Regarding overall fatty acids, *m. gluteus superficialis* of Saanen goat had the highest content of total SFAs and UFAs compared to the other examined goats, but, at the same time, Balkan goat meat was determined to have the highest content of PUFAs. Balkan goat meat had more OFAs, but essential fatty acids (EFAs) were more prominent in Alpine goat meat. Ratios of PUFAs and SFAs are recommended to be higher than 0.4 (Wood et al., 2008), and in this trial they were higher than the recommended level for all examined breeds. Calculated nutritional value was the highest for Saanen goat meat. Calculated ratios were in-line with findings of some other authors (Peña et al., 2009; Solaiman et al., 2011). Results presented here are not in-line with Özcan et al. (2015), which may be the consequence of different experimental approaches, as well as different breeds and ages of goats included in the trial.

High amounts of unsaturated and particularly polyunsaturated fatty acids are thought to enhance the nutritional and health-promoting value of meat. However, unsaturated fatty acids are more susceptible to oxidation and thus can have an influence on meat sensory characteristics and, furthermore, can cause toxic compound synthesis (Daszkiewicz and Mesinger, 2018).

The most common group of compounds identified in meat from all three breeds was aldehyde, with hexanal being predominant among the volatiles (up to 16.12 $µg\;{\mathrm{kg}}^{-1}$). Other volatile compounds were determined in the following order: ketones > alcohols > alkanes > organic acids > alkenes > aromatic hydrocarbons > heterocyclic compounds.

Aldehydes in general are major sources of volatile fractions obtained from domestic ruminant meat (Vasta and Priolo, 2006; Villalobos-Delgado et al., 2014; Ivanović et al., 2016). Within the group, linear aldehydes that are produced in fat oxidative degradation were detected with the exception of benzaldehyde (Belitz and Grosch, 1987; Ripoll et al., 2019). Hexanal, predominant in the examined samples, is mainly derived from linoleic and arachidonic acids (Martin et al., 2002). Measured concentrations of those fatty acids in goat meat samples differed significantly between breeds, as well as hexanal concentrations. Aldehydes have a low aroma threshold and an intense and specific aroma, which can make them important contributors to the aromatic meat profile (Madruga et al., 2009). They have a greasy, green grass and apple flavor, especially hexanal, which is described as having a delicate and grassy fragrance related to the smell of newly mown grass but also possibly having the bad smell of green beans and a rancid aroma when present in high concentrations (Frank et al., 2017; Chen et al., 2019). Aldehyde content is determined by fatty acid and protein content in meat and varies among breeds. That fact points out the impact of breed and genotype on the volatile profile of meat. The determined aldehyde levels in Balkan goat meat were in agreement with the results obtained in previous studies of meat from Balkan goats reared in a mountainous region (Ivanović et al., 2016).

Aldehydes are believed to form as a result of lipid oxidation and protein degradation, while ketones generally correlate with the type of diet (Ivanović et al., 2016). Among ketones, the highest determined concentration had 2-butanone. 2-Ketones are significant contributors to meat aroma and flavor because they have a peculiar aroma that is described as having ethereal, buttery and spicy notes or blue cheese notes (Creuly et al., 1992; Lecanu et al., 2002). Determined levels of 2,3-octanedione fit with the suggestion that it occurs in higher amounts in meat from grass-fed animals (Vasta and Priolo, 2006). Sivadier et al. (2010) suggest that 2,3-octanedione may serve as a biomarker confirming that meat originates from animals feeding in pastures (Kosowska et al., 2017).

Alcohols are formed as products of the oxidation of lipids or their oxidation products, like hexanal or heptanal (Kosowska et al., 2017). Furthermore, alcohol formation is attributed to microorganisms' activity, like 3-methyl 1-butanol being formed in the degradation of amino acids (Muriel et al., 2004). Alcohols have herbaceous, woody and fatty notes (Lorenzo et al., 2013). The determined levels of aldehydes in the current study that can be precursors for alcohol synthesis were significantly different between breeds, as well as levels of alcohols, which supports the breed's impact on volatile compounds in meat.

Only small amounts of organic acids in meat were detected in the current study, although they are responsible for the distinctive taste of goat meat. Organic acids with less than six carbon atoms (short chain acids) are considered significant contributors to the volatile profile of meat because of their specific odors – vinegar, cheese or cucumber – as well as their low threshold values (Lorenzo et al., 2013). A pungent and sour odor note is attributed to acetic acid (Kosowska et al., 2017).

From the group of alkanes, octane was the most prevalent followed by hexane. Except for octane, the determined amount of alkanes was at the level of 0.2 $µg\;{\mathrm{kg}}^{-1}$. It can be seen that alkanes did not have a significant influence on goat meat taste and aroma due to their high threshold values (Drumm and Spanier, 1991).

The presence of aromatic hydrocarbons, alkanes and heterocyclic compounds was marginal, but they may have synergistic effects with other compounds in the formation of meat aroma and taste.

The color results obtained differed from our previous investigations regarding Balkan and Bunte Deutsche Edelziege goats (Ivanović et al., 2011) and goat meat quality (Ivanović et al., 2014). On average, meat from Saanen goat was lighter compared to the other two examined breeds. Furthermore, the same breed had higher redness and yellowness parameters compared to the other breeds and significantly higher compared to Balkan goat (P<0.05).

Meat color may be affected by numerous factors, including type/breed, structure and the ratio of intramuscular fat (Ivanović and Pavlović, 2015), which may be the cause of color differences in the current study. The color of meat may correlate with the content of intramuscular fat. In the current study, the intramuscular fat content and fatty acid profile differed significantly between breeds. Balkan goat had the lowest total fat content, followed by Alpine and Saanen goats. In accordance with that, color parameters were the lowest for Balkan goat and significantly lower compared to the other two examined breeds. Although there were not significant differences between Alpine and Saanen goat meats for L*, a* and b* values, they differed in the same order for total fat content measured, which may be the cause of color differences between the examined breeds. Similar to those findings, Peña et al. (2009) and Dhanda et al. (2003) proved the influence of breed on meat color. On the other hand, Madruga et al. (2008) show significant differences (P<0.05) in the values for the a* parameter of meat color between breeds but not for the L* and b* parameters.

Balkan goat meat was more sensorially acceptable than Saanen goat meat overall. The results of the current study have slight differences in the sensory evaluation of the color and the odor of the examined samples, which are both factors that affect the overall acceptability of meat. That is consistent with the findings of other authors (Peña et al., 2009; Ding et al., 2010). However, sensory evaluation is very difficult to compare between studies as the meat evaluated derives from goats reared in completely different conditions (climate, feed and growing conditions). Furthermore, different techniques in sensory evaluation are applied.

## 5 Conclusions

In comparing the results of analyzed meat quality parameters of meat from Balkan, Alpine and Saanen goats, significant differences were measured in moisture, total fats, proteins, ash, instrumentally defined color and the sensory evaluation of meat. Furthermore, differences were determined in the fatty acids and volatile profile of meat. Alpha-linolenic (n-3 FA) and linolelaidic acids were found in higher percentages in Balkan goat meat, while Alpine goat meat contained the highest amounts of linoleic acid among the three goat breeds. Balkan goat meat contained fewer volatile substances than meat from Alpine and Saanen goats. The overall sensory acceptability was ranked the best for Balkan goat meat. From the presented results, it can be concluded that from meat obtained from indigenous breeds, the Balkan goat had favorable nutritional and health benefits, particularly if the consumer's demand is lean meat.

The results obtained in this study suggest breed has an impact on the quality of goat meat and, therefore, potentially on the quality of goat meat products. However, the current study is not without limitations. First of all, some fatty acids and volatile compounds were not identified, indicating that further research is required. Furthermore, the evaluation of fresh meat was conducted, which does not reflect the conditions in which goat meat is routinely processed; thus, studies should be carried out on such meat products as well. Finally, it would have been helpful to carry out studies which include several different breeds.

## Data Availability

The underlying research data can be obtained from the corresponding author, who can be reached by mail: majaspavlovic@gmail.com.
